# On the Treatment of Poisoning by Arsenic

**Published:** 1854-03

**Authors:** W. S. Barker

**Affiliations:** Beloit, Wis.


ART. II.—*On the Treatment of Poisoning by Arsenic.* By Dr. W. S. BARKER, Beloit, Wis.

CASE. A child aged six years, son of Wm. Dyer, of this village, was poisoned by arsenic, Oct. 2d, 1853. The mother of the boy saw some rats in the house, and thought she would destroy them with poison. She put about a table-spoonful of arsenic on a plate, and being called to another part of the house, left it on a table in the parlor. Returning to the room after a few minutes absence, she saw the child leisurely devouring the poison. His mouth was filled with it, and the tongue was covered from tip to base. She made him spit out much of it, but the boy himself said he had already swallowed more than he had in his mouth at the time she detected him. Though it would be impossible to say with exactness, I should think he had swallowed not less than 30 or 40 grains. I was called at 8½ P. M., 30 minutes after the poison was taken.

*Symptoms:* When I arrived the patient complained of severe burning pain at the precordia; nausea; respiration difficult; pulse small, frequent, (137) and very irregular, and an unusual heat over the whole body.

*Treatment:* Emetic of sulphate of zinc, which acted after copious draughts of milk. He had drank water before I saw him, but had not vomited. I then filled a quart bowl with magnesia and milk, a part of which was taken and immediately rejected. The dose was repeated in like manner, until all was drank, though but little was retained in the stomach, and no benefit was perceptible.

At 10 P. M. the child seemed rapidly growing worse. Countenance anxious; great soreness in the stomach, tremor of the limbs, retchings; in short unmistakable symptoms of arsenical poisoning. I then prepared and administered the following:

Gum. Arabic Mucilage ℥vj.

Ferri Carb. ʒij.

Cretae pptae. ʒj.

He took 3 such doses. Soon after the first dose he seemed better. The pain was much less, and the throat became moist. After the second he felt quite easy, tongue moist, skin not so hot, pulse 85 and more full and steady.

After the third dose all signs of poisoning ceased. He went to sleep and slept well for six hours. In two days he was as well as usual, and has since continued in perfect health.

*Remarks.* Judging from the apparent effects of the carbonate of iron in this case, I firmly believe the recovery of the patient was owing to its timely administration. I think that notwithstanding the other means resorted to, he would probably have died without this antidote.

I presume it is generally known by the profession, that in 1834 Dr. Bunsen, a German Chemist, proposed the use of the hydrated peroxyde of Iron in a moist state, as an antidote to arsenic. A case was soon after treated with it successfully; an account of which was published in the Revue Medicale.

Many experiments were afterwards tried on the inferior animals, the result of which sustained Mr. Bunsen's opinion.

Numerous trials, chemical and physiological, were then made to test the efficacy of the dry peroxyde of iron, and its antidotal power was found to be considerable when the dose of the poison had not exceeded four or five grains.

Consequently the hydrated peroxyde of iron held the supremacy and was recommended by M. Orfila and other eminent toxicologists.

No one therefore can question the value of this substance in arsenical poison. But although there is no objection to the article itself, it, like many other good and desirable things, is frequently difficult to procure.

In fact, in most places, it is not to be had at all. A practical chemist only can prepare it, and but few physicians in general practice, particularly those residing in country towns and villages, would attempt it.

Moreover it will not keep for a length of time, but must be freshly prepared.

The proeess of preparing it is very tedious, requiring of even an experienced chemist four or five hours time. To be sure, the dry peroxyde can be prepared with less trouble, but even this takes more time than a practitioner would like to spare after he is called to treat a case of poisoning, knowing as he must, that the sooner after the ingestion of the poison the antidote is given, the greater will be the probability of its efficacy. What, then, can he do? If he has no faith in magnesia or charcoal in such cases, and doubts whether good effects would result from bleeding, or stimulants, he would probably either trust his patients to chance or commence the preparation of the oxyde of iron. But let us stop for a moment and inquire, concerning the modus operandi of this antidote. It unites with the arsenic and forms an inert, insoluble compound, and "there is reason to believe that if a complete union could take place in the stomach *soon after the poison had been swallowed*, and before it has produced its deliterious effects, life might be saved.

But the difficulty is, that during the time necessary to prepare the peroxyde. a considerable quantity of the poison is absorbed and beyond the reach of a chemical antidote. Therefore, I would suggest that in all cases af poisoning by arsenic it would be well, after expelling as much as possible of the metal by vomiting, to administer the carbonate of iron.

In the case the history of which I have just given, the poison was taken into the mouth dry, and some water drank soon after, which, of course, promoted its solubility, and favored its absorption. The plan of treatment generally adopted, did not in the least mitigate the symptoms, which were very severe. But after taking the iron, the improvement was marked, progressive and permanent.

At the time this case occurred in my practice, I was not aware that the carbonate of iron had even been used as an antidote to arsenious acid. I have since read in the Medico-Chirurgical Review an account of two cases, which were under the observation and treatment of an experienced English Surgeon, P. L. Serph, of Welshpool.

He gave carb. ferri in both cases, and attributed the recovery of his patients to it. His first case was a boy twelve years of age, who swallowed by mistake a tea-cupful of a solution of white oxyde of arsenic, prepared by a member of the family to cure the itch. Mr. Serph thought the solution contained a scruple of the oxyde.

Case second, was an adult who had washed his body and limbs in a strong solution of arsenic, recommended by a *friend* for some malady with which he thought himself afflicted. The symptoms in both cases were severe, threatening the extinction of life, but yielded rapidly after the administration of carb. of iron in large doses.

As the use of this antidote, if I may be allowed to call it such in these three cases the only instances of arsenical poisoning in which I have known of its use, was attended with entire success, I think, I may with propriety, call the attention of members of the profession to the subject.

The want of a reliable antidote to arsenic, that could be had at a moment's warning and administered without delay, has been much deplored.

Poisoning by arsenic is not so infrequent an occurrence as some may suppose. Where self destruction is attempted by poisoning, arsenic is generally selected. "Of 543 fatal cases of poisoning, upon which coroners' inquests were held in England during two years, death was caused in 186 cases by arsenic. Of 288 cases in France, 196 were caused by arsenical compounds."

I will conclude this article, which is already longer than I designed writing when I began, by expressing the wish, that its publication may induce others to make use of this preparation of iron, and that it may prove as valuable as in the cases already cited.

